# The Impact of Epigenetic Signatures on Amniotic Fluid Stem Cell Fate

**DOI:** 10.1155/2018/4274518

**Published:** 2018-11-25

**Authors:** Daniela Di Tizio, Alessandra Di Serafino, Prabin Upadhyaya, Luca Sorino, Liborio Stuppia, Ivana Antonucci

**Affiliations:** Department of Psychological, Health and Territorial Sciences, School of Medicine and Health Sciences, “G. d'Annunzio” University, Chieti-Pescara, Italy

## Abstract

Epigenetic modifications play a significant role in determining the fate of stem cells and in directing the differentiation into multiple lineages. Current evidence indicates that mechanisms involved in chromatin regulation are essential for maintaining stable cell identities. There is a tight correlation among DNA methylation, histone modifications, and small noncoding RNAs during the epigenetic control of stem cells' differentiation; however, to date, the precise mechanism is still not clear. In this context, amniotic fluid stem cells (AFSCs) represent an interesting model due to their unique features and the possible advantages of their use in regenerative medicine. Recent studies have elucidated epigenetic profiles involved in AFSCs' lineage commitment and differentiation. In order to use these cells effectively for therapeutic purposes, it is necessary to understand the basis of multiple-lineage potential and elaborate in detail how cell fate decisions are made and memorized. The present review summarizes the most recent findings on epigenetic mechanisms of AFSCs with a focus on DNA methylation, histone modifications, and microRNAs (miRNAs) and addresses how their unique signatures contribute to lineage-specific differentiation.

## 1. Introduction

**Figure 1:**
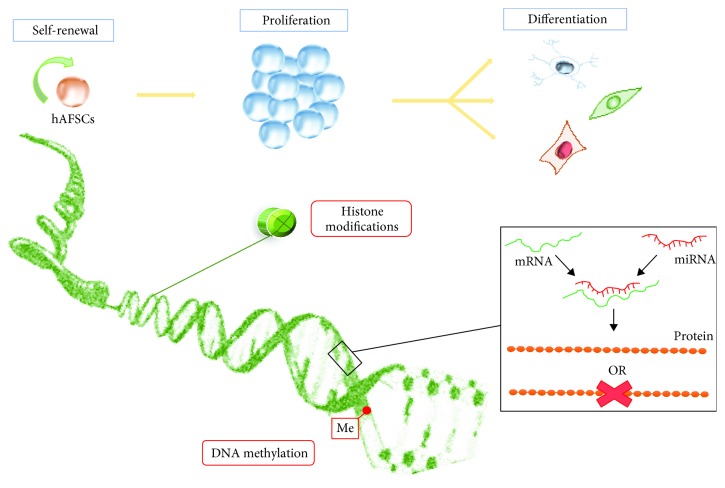
Epigenetic mechanisms implicated in the self-renewal, differentiation, and proliferation of hAFCS.

One of the fascinating fields of modern medicine is epigenetics, a new discipline that studies heritable changes in gene expression not related to alterations of DNA sequence. The role of epigenetic mechanisms in cell fate determination is widely reported in scientific literature [1–3]. Alterations of the epigenetic networks may lead to the loss of self-renewal capacity and lineage commitment anomalies resulting in dysregulation of the differentiation process of intermediate progenitors [4–6]. The full complexity of epigenetic machinery is only partially known, and the major molecules involved in regulating gene expressions (DNA methylation, histone modifications, and small noncoding RNAs) can contribute to cellular plasticity and influence the transition from a pluripotent state to a multipotent state, in the context of landscape models [7, 8]. During the differentiation process, DNA methylation plays an important role in silencing pluripotency-related genes and activating tissue-specific genes, thus producing a cellular memory that defines the cell fate commitment. Recent findings have shown that gene regulation at posttranscriptional levels influences the stemness and biologic characteristics of stem cells [9–11]. Current studies on miRNAs have revealed a signature that is characteristic for the state of embryonic stem cells (ESCs) [12–14]. Indeed, some miRNA clusters act as cell cycle moderators increasing ESC proliferation due to the promoted transition from the G phase to S1 phase [15]. Moreover, miR-291-3p, miR-294, and miR-295 families regulate core pluripotency transcription factors (Oct4, Nanog, and Sox2) and promote somatic reprogramming through signaling cascades as mesenchymal-to-epithelial changes necessary for colony formation, a reduction of differentiation potential, and an inhibition of cellular senescence [16]. Another significant epigenetic process is dynamic chromatin remodeling associated with cell fate decisions. The advent of high-throughput next-generation sequencing (NGS) has increased new insights regarding the function and activity of histones. For instance, acetylation of histone residues generally is associated with gene activation, while methylation can have both activating and repressive roles [17]. Although every stem cell has a unique epigenetic signature, a common message derives from all these studies: cells need both the DNA “hardware” and the epigenetic “software” in order to respond to the requirement of the tissues [18]. A better understanding of all these complicated processes is necessary to choose a safe source of stem cells to be used in the clinical context. In view of this evidence, AFSCs represent a novel and alternative resource for regenerative medicine, due to their high renewal capacity and the plasticity intermediate between embryonic and adult stem cell types [19–21]. These cells are obtained during the process of amniocentesis (around the 16th week of pregnancy) and contain a heterogeneous population of cell types originating from embryonic and extraembryonic tissues. For this reason, they could provide an *in vitro* model for studying epigenetic regulation in early human development. A growing body of evidence has demonstrated that the ability of differentiation of AFSCs [22–24] is the result of a complex and dynamic network of transcriptional factors, and certainly the epigenetic mechanisms play a central role in activation and/or repression of tissue-specific genes (see Figure 1). To date, how these cells acquire new fates is still unknown and many processes governing their biology should be explored in order to add new knowledge to this complicated epigenetic puzzle. This review summarizes recent findings on epigenetic signatures of AFSCs related to lineage commitment and plasticity, highlighting their potential into clinical practice.

## 2. Epigenetic Changes in AFSC Reprogramming

**Figure 2:**
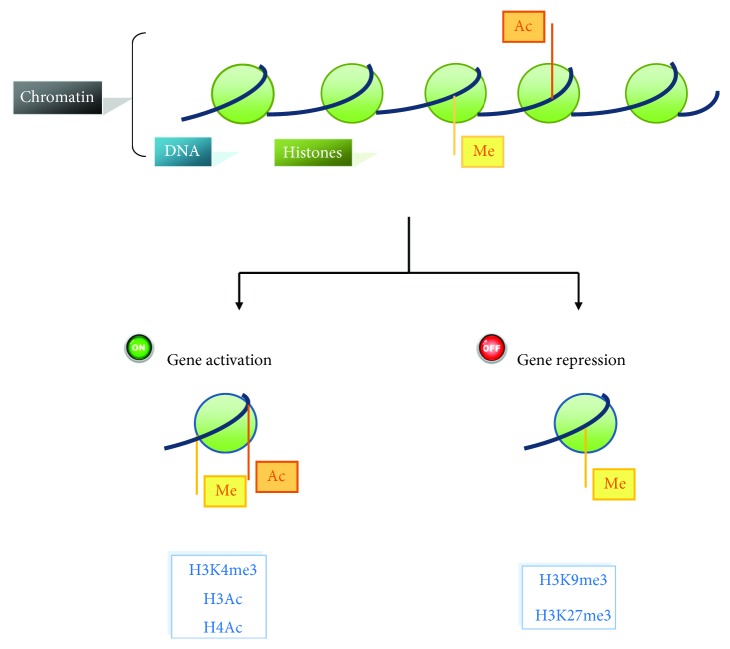
Epigenetic modifications induced by methylation and acetylation of H3 and H4.

In recent years, AFSCs have emerged as an alternative source for cell reprogramming and regenerative medicine since they display an “intermediate phenotype” between embryonic and adult stem cells, in addition to an active telomerase activity and a high degree of plasticity [23, 24]. They are easily achievable, show high proliferation rate and negligible immunogenicity, lack tumorigenicity, and demonstrate no ethical concerns [25]. The identification of the “best” cell type and the right protocol that guarantee the maximum efficiency during the generation of induced pluripotent stem cells (iPSCs) represents an important milestone in the knowledge of human diseases. In iPSCs, epigenetic modifications cause the change in the expression of many genes, suggesting that such modifications may directly determine the fate of the stem cells [26]. In light of these considerations, several studies have documented the capacity of human amniotic fluid stem cells (hAFSCs) to generate iPSCs using defined protocols. The application of hAFSCs in the reprogramming process has been pioneered by Li and colleagues in 2009 when they demonstrated for the first time an efficient generation of human amniotic fluid-derived induced pluripotent stem cells (hAFiPSCs). In this study, the pluripotency was induced through retroviral delivery of four human transcription factors (Oct4/Sox2/KLF4/c-Myc) and all three germ layers are found in embryoid bodies and teratomas, respectively [27]. More detailed molecular studies demonstrated the ability of hAFiPSCs to differentiate into the extraembryonic trophoblast lineage, and comparative transcriptome analysis with human embryonic stem cell (hESC) lines revealed a subset of genes expressed in all pluripotent cell lines including common self-renewal and pluripotency-associated gene regulatory network upon cellular reprogramming [28, 29]. For this reason, AFSCs could be easier than somatic cells to reprogram to pluripotency because of their similarity in transcriptional and epigenetic states with embryonic cell types. Pluripotent stem cells are characterized by a unique epigenetic profile enhanced for active chromatin modifications, histone acetylation, and hypomethylated DNA [30]. In particular, the initial phase of reprogramming is strictly related to an open chromatin state and active transcription, such as acetylation of histones H3 and H4 [31]. Similarly, trimethylation of the histone 3 at the fourth lysine residue (H3K4me3) is considered as a potent marker of active chromatin state [32] and is associated with DNA hypomethylation in many genomic loci in iPSCs [31, 33, 34]. Despite the presence of the activating histone marks, H3K9 methylation is considered as a repressive marker and during reprogramming it is necessary to repress H3K9 methyltransferases (Ehmt2, Setdb1, and Suv39h1/2) in order to open the chromatin [35–37]. Generally, modifications like methylation at of H3K9 and H3K27 residues are associated with compact chromatin and transcriptional repression, often found in silent gene loci (see Figure 2) [38].

Although the generation of a nonviral iPSC remains an important challenge, it has been reported for the first time that human first-trimester AFSCs can be fully reprogrammed to pluripotency without ectopic factors, by culture on Matrigel in a hESC medium supplemented with the histone deacetylase inhibitor (HDACi) valproic acid (VPA) [39]. These data identify an ideal human cell source for rapid and efficient generation of iPSCs and the possibility to study *in vitro* genetic disorders associated with pregnancy. In fact, AFSCs can be used to generate patient-specific pluripotent cells for the use in regenerative medicine, pharmaceutical screening, and disease modeling. In some cases, prenatal diagnosis allows isolation of hAFSCs from fetuses with chromosomal anomalies and their use could provide a unique opportunity for the modeling of genetic diseases and to predict the outcome of several pathologies [40]. In this regard, in the same year, interesting results have clearly demonstrated that *β*-thalassemia patient-specific autologous induced pluripotent stem cells can be rapidly and efficiently generated from cultured terminally differentiated AFSCs using a single excisable lentiviral stem cell cassette [41]. In addition, AFSCs with trisomy 21 have been used to obtain iPSCs in modeling of Down syndrome and hence impaired neurogenesis has been observed [42]. However, in all these studies, virus-based integrating reprogramming approach was used but the risk of insertional mutagenesis into the cell genome is very high. To address this issue, numerous strategies constituted by the PiggyBac (PB) transposon system and chemically defined culture have been developed [43, 44] for eventual clinical translation of hAFiPSCs into cell therapies. In this regard, one study provides compelling evidence on the possibility to generate a population of beating amniotic fluid-derived cardiomyocytes (AF-CMs) after Sendai virus reprogramming towards pluripotency [45]. Similar data were confirmed by other groups that have focused their attention on possibility to obtain functional cardiomyocytes from iPSC reprogramming using nonviral methods [46]. Taken together, these findings provide evidences that amniotic fluid (AF) could represent an ideal source for autologous cells for the treatment of neonatal congenital heart defects and neurodegenerative diseases. In particular, Qin et al. have evaluated AFiPSC membrane-derived vesicles for repairing of cerebral ischemic damage and in addition to it, they have demonstrated the generation of AFiPSCs with ectopic expression of the transcription factor Oct4 alone [47]. Recently, the suitability of the AFSCs in reprogramming application for the generation of iPSCs and subsequent differentiation into haematopoietic and neural lineages was identified [48]. In line with these promising findings, there is an increasing interest in the possibility of cultivating AFSCs from fetuses affected by genetic diseases in order to study the processes of tissue differentiation in pathological conditions [23, 40] and to generate disease modeling by applying reprogramming technologies.

## 3. MicroRNA Profiling of hAFSCs: Key Regulators of Self-Renewal, Differentiation, and Proliferation

**Figure 3:**
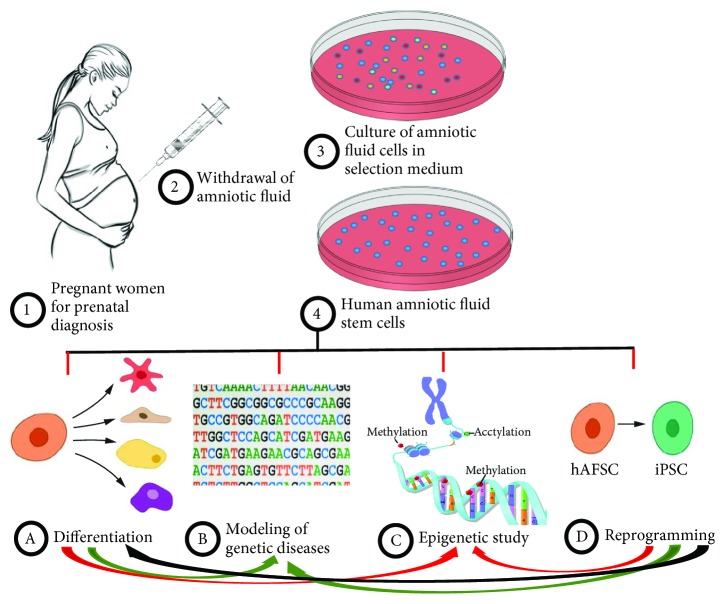
Several applications of hAFSCs for different biological and molecular studies.

MicroRNAs or miRNAs belong to a class of highly conserved small noncoding RNAs (18–25 nucleotides in length) that play a key role in posttranscriptional gene regulation in many organisms [49, 50]. In mammals, miRNAs are also involved in the early maturation of embryos, stem cell differentiation, and apoptosis [51–53]. The outstanding roles of miRNAs in stem cells have been investigated in a wide range of biological processes, including self-renewal, differentiation, and proliferation [54]. Since miRNAs can repress the translation of many mRNA targets, they are ideal candidates to regulate cell fates [55]. A large body of evidences suggests that miRNAs in the amniotic fluid (AF) act as modulators to regulate the expression of specific genes during fetal development [56, 57]. It is important to emphasize that the components of AF are mainly derived from the fetus and the presence of specific microRNAs may be able to give important information on fetal development, physiology, and pathology during pregnancy [58–60]. In this scenario, growing interest has focused on epigenetic properties of hAFSCs in order to identify new markers of stemness, self-renewal, and differentiation. Current literature data show that miRNAs play a crucial role in the fate of hAFSCs and in particular they are involved in controlling the WNT signaling, MAPK signaling, and TGF-*β* signaling pathways [61]. In addition, a first characterization of the regenerative potential of the hAFSC-derived extracellular vesicles (EV) has identified specific miRNAs involved in their paracrine effects [62]. These results are coherent with previous findings, which reported that specific miRNAs (miR-146a and miR-10a) obtained from hAFSC-derived exosomes have a therapeutic effect in a mouse model of cyclophosphamide- (CTx-) induced premature ovarian failure (POF) [63]. Finally, the temporal analysis of the hAFSCs between passages 1 and 15 showed significant variation in the expression of multiple genes and miRNAs. These changes are mainly related with the downregulation of TP53 and the increased expression of hsa-miR-125a, which could act as indicators of the state of proliferative capacity and stemness [64]. Taken together, these data provide the possibility of a novel clinical cell-free therapeutic strategy for the treatment of several human diseases. Another important aspect is the involvement of small RNAs in mammalian cell differentiation. In fact, these molecules accelerate the change of cell state into progenitor cell lineages [65]. Emerging evidences demonstrate that in *in vivo*, the microenvironment decides the fate of multipotent, pluripotent, or totipotent cells, towards the commitment into a specific lineage [66, 67]. However, there are very less shreds of evidences explaining how the microenvironment precisely changes the character and properties of the cells, changing it from one morphological state of stem cells to another morphological state of differentiated cells. With the advances in *in vitro*, cell culture and molecular techniques, a number of factors responsible for cellular differentiation have been elucidated. With these discoveries, the most frequent questions we come across are how these differentiation factors actually carry out differentiation and what happens to the cells after they get differentiated. It is documented that hAFSCs have the remarkable potential to develop into many different cell types [42, 68, 69], but to date there is not much information regarding epigenetic changes associated with their multilineage differentiation capability. miRNA expression studies involved in the differentiation of hAFSCs have been pioneered by two researchers in 2014 when they demonstrated that these cells displayed an appreciable ability to differentiate towards osteogenic and chondrogenic lineage but failed adipogenesis, as proved by expression of miR-21, mainly implicated in osteogenesis differentiation [70, 71] (see Figure 3). These data were also confirmed by Gholizadeh-Ghaleh Aziz et al. that highlighted the possible role of miR-302a and miR-Let7g in the interaction with Nanog and POU5F1 and involvement in two differentiation processes of adipogenesis and osteogenesis [72]. Finally, as stated in the study of Glemžaitė and Navakauskienė, osteogenic differentiation of hAFSCs is mediated by chromatin-modifying enzymes, histone modifications, and specific microRNA expression [73] (see Table 1). These observations suggest the importance of miRNAs in the regulation of AFSC fate and reinforce the necessity of further studies to investigate their potential therapeutic applications in the field of regenerative medicine.

## 4. Epigenetic Markers of Fetus-Affected Pregnancies

**Figure 4:**
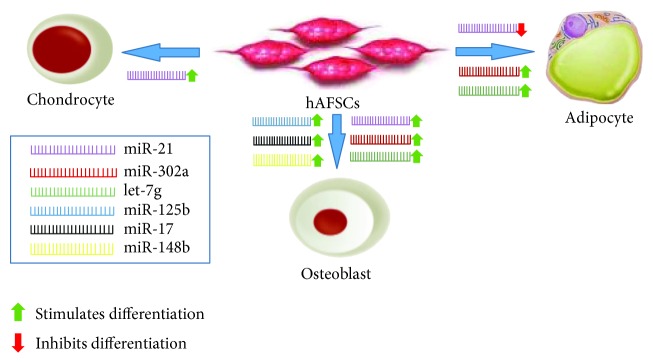
The role of miRNAs in hAFSCs during chondrogenic, osteogenic, and adipogenic differentiation.

Current research has focused on the role of epigenetic processes in embryo development and growth, in particular on the enhanced susceptibility to fetal diseases. In recent years, there is an explosion of research in the field of “environmental epigenomics” based on the relationships between environmental exposures, epigenetics, and human health and diseases [74, 75]. The prenatal period represents a critical period of development during which adverse conditions and environmental exposures may influence offspring health and behavioral outcomes [76, 77]. One of the most intriguing aspects of epigenetic research is the concept of transgenerational inheritance. This phenomenon explains that some epigenetic changes occurring in the germline have the potential to be transmitted to the offspring. In support of this notion, it has been recently shown that AFSCs share several features with primordial germ cells (PGCs) [24], thus suggesting their use as an efficient handy tool to study human gametogenesis and understand the diseases caused by epigenetic alterations endangering transgenerational inheritance [40]. In some cases, prenatal diagnosis allows the detection of genetic diseases and the use of AFSCs could provide a very interesting model to understand the molecular basis of pathologies and to identify new therapeutic approaches [23, 40]. In this scenario, in 2013, Tsurubuchi et al. published data describing the presence of novel biomarkers for early detection of neural tube defects (NTDs) in human fetuses. This study examined the expression of epigenetic histone marks in hAFSCs cultured from women pregnant with normal or NTD-affected fetuses. In detail, stem cells obtained from the anencephaly-affected pregnancy showed higher levels of H3K4me2/me3 and H3K27me2/me3 together with lower levels of H3K9ac and H3K18ac in cultures [78]. This is a first step in the identification of novel biomarkers for NTDs, and in the future, these epigenetic marks could be tested in the blood of pregnant women in order to develop specific treatments for in utero closure of these defects. In addition, epigenetic factors are implicated in senescence process of AFSCs and a diversity of proliferation and senescence of cells from AF of normal gestation and fetus abnormalities has recently been reported. The two sample groups showed different dynamic changes in chromatin structure during cell senescence. In particular, it was reported that stem cells derived from AF with fetus abnormalities led to senescence in rather early passages (from 5 to 8) and showed the changes in morphological and senescence associated with repressive histone modifications (H3K9me3 and H3K27me3) [79]. In 2017, the same researchers performed further experiments to better understand the epigenetic environment in terms of histone modifications in hAFSC cultures in normal and diseased gestation conditions. The comparison of healthy and pathological samples revealed that two distinct cultures especially with regard to the proliferation potential of hAFSCs from some donors with genetic or multifactorial fetal diseases displayed distinct growth, the alterations in global DNA methylation, changes in the pattern of acetylated histones H3 and H4, and dysregulation of both methylated histones H3K27 and H3K9 [80]. Considering the fact that to date epigenetic mechanisms in abnormal prenatal conditions are not well known, these results lay the foundation for identifying novel biomarkers for human diseases in the serum of pregnant women (see Figure 4). A very interesting recent paper described the presence of significantly dysregulated miRNAs in AFSCs from pregnant women carrying fetuses with Down syndrome (DS). In the future, these findings may provide valuable insights regarding the use of miRNAs in the prenatal diagnosis of DS (see Table 2). Nevertheless, the association between miRNAs encoded by chromosome 21 and the various phenotypes of DS should be further investigated before their diagnostic application [81].

## 5. Status of Genomic Imprinting of Human AFSCs

The genomic imprinting represents a non-Mendelian heredity model and is a process of “marking” of some genes preferentially expressed from a single parental allele. Imprinted genes exert broad roles and influence fetal growth, pluripotency, differentiation, and behavior [82]. Scientific evidence suggests that dysregulation of imprinted gene dosage can be involved with tumorigenesis and altered cell differentiation ability [83, 84]. In humans, abnormal imprinting patterns are associated with congenital disorders, including Beckwith–Wiedemann, Silver–Russell, Angelman, and Prader–Willi syndromes [85, 86]. Several studies have previously tested the status of imprinted genes in hESCs, suggesting that SNRPN, IPW, and KCNQ1OT1 were highly stable and insensitive to epigenetic perturbations. In contrast, H19, IGF2, and MEG3 were more variable and could provide an indication of epigenetic status [87–89]. These evidences confirm that hESC lines expressing monoallelic gene after differentiation may be better suited to therapeutic use than lines that show variable expression. There is a growing attention in the study of alterations of imprinted genes in hAFSCs during *in vitro* cell culture. Unfortunately, until now, there is no extensive literature documenting the changes in expression of genomic imprinting of these cells. In 2012, the first study on alterations of imprinted genes (H19, SNRPN, and KCNQ1OT1) in hAFSCs was performed by Peng et al. In particular, it was observed that during *in vitro* hAFSC culture, there are hypermethylation of H19 and KCNQ1OT1 and variable DNA methylation patterns in the SNRPN gene [90]. These results were in agreement with the study by Phermthai et al. which demonstrated the aberrant expression of the IGF2 and H19 genes in late passages of AFSCs [91]. On the basis of these preliminary observations, it can be assumed that the epigenetic instability correlates with the loss of differentiation potential in late passages of AFSCs, and due to this reason, the therapeutic use of these cells should be limited to the 8^th^ passages. Most recently, epigenetic analysis showed that AF is one of the most interesting sources for medical therapy because AFSCs display high genomic stability and epigenetic fidelity [84]. These data reinforce the necessity of further studies to investigate the therapeutic potential of hAFSCs and support their use in the clinical therapy.

## 6. Conclusions

The surprising picture that has emerged in the past few years is the therapeutic potential of hAFSCs in the field of regenerative medicine and the possibility to develop novel strategies against a wide range of human disorders. The progresses of preclinical experiments are encouraging but important aspects should be explored in order to support the use of hAFSCs in the clinical application in the near future. In this scenario, the molecular characterization of these cells has a relevant importance as they can be used in regenerative medicine. In this review, we have focused on the current knowledge concerning the role of epigenetic changes in proliferation, differentiation of hAFSCs, and biomarkers involved in diseased gestation conditions. The potential use of AFSCs for tissue regeneration was shown to be successful in animal models, and elucidating their differentiation process is fundamental to avoid undesired side effects in future clinical applications. To date, little is known on histone and DNA methylation modifications of hAFSCs but some data support the notion that epigenetic machinery by histone modifications and chromatin remodeling is involved during osteogenic and adipogenic differentiation. Another interesting feature is represented by the possibility to cultivate these cells from affected fetuses in order to study the molecular mechanism underlying the development of congenital malformation. In this aspect, hAFSCs could represent an interesting alternative to iPSCs for identifying epigenetic marks in diseased gestation. On the basis of preliminary observations, these markers could be used for early detection and future identification of abnormal prenatal conditions in amniotic fluid and maternal serum. In consideration of data described in the present review, it is possible to conclude that epigenetic modifications are involved in the following:

- Self-renewal and differentiation of AFSCs
- The generation of iPSCs from human AFSCs
- Fetal cell cultures associated with the genetic disease
- Studies of transgenerational inheritance

Finally, further investigations should be conducted to define a better comprehension of “epigenetic landscape” of hAFSCs in order to expedite the progress of stem cell-based therapeutics in regenerative medicine.

## Figures and Tables

**Table 1:** Epigenetic regulation of osteogenic and adipogenic differentiation of AFSCs.

Epigenetic changes	Effect of alteration	Finding	Reference
miR-125b	Overexpression during osteoblastic differentiation	Does not influence levels of Runx2, osteopontin, and ALPL gene expression	[92]
miR-21	Overexpression during osteoblastic and adipogenic differentiation	Accelerates osteogenesis and decelerates adipogenesis	[70]
miR-302a and miR-Let7g	Upregulation of miR-302a and downregulation of miR-Let7g in AFSCs	Interact with Nanog and POUSF1 and are involved in adipogenesis and osteogenesis differentiation	[72]
H3K9ac, H4 hyperAc, H3K27me3, miR-17, and miR-148b	Upregulation of acetylation of H3K9 and H4, reduction of H3K27me3, and upregulation of miR-17 and miR-148b in osteogenic differentiation	Osteogenic differentiation is related to histone modifications and specific microRNA expression	[73]

**Table 2:** Associations between epigenetic modifications and diseased gestation condition.

Disease	Epigenetic marks	Alteration	Reference
Neural tube defects	Histone	(i) Myelomeningocele (a) High levels of H3K4me2, H3K4me3, H3K27me2, and H3K27me3; (b) Low levels of KDM6B; (c) Decreased levels of H3K9ac, H3K18ac, and Gcn5. (ii) Anencephalic (a) increased levels of H3K27me3, H3K9Ac, H3K18Ac, and Gcn5.	[78]
Fetus-affected pregnancies	Global DNA methylation histone	Alterations in global DNA methylation, H4K16ac, H3K9ac, and H3K14ac and dysregulation of H3K9me2/me3 and H3K27me3	[80]
Down syndrome (DS)	MicroRNA	High levels of miR-125b-2, miR-155, and miR-3156 in pregnant women carrying fetuses with DS	[81]
